# Protein Phosphorylation Profiling Using an *In Situ* Proximity Ligation Assay: Phosphorylation of AURKA-Elicited EGFR-Thr654 and EGFR-Ser1046 in Lung Cancer Cells

**DOI:** 10.1371/journal.pone.0055657

**Published:** 2013-03-08

**Authors:** Tzu-Chi Chen, Yu-Wen Liu, Yei-Hsuan Huang, Yi-Chen Yeh, Teh-Ying Chou, Yu-Chung Wu, Chun-Chi Wu, Yi-Rong Chen, Hui-Chuan Cheng, Pei-Jung Lu, Jin-Mei Lai, Chi-Ying F. Huang

**Affiliations:** 1 Institute of Clinical Medicine, National Yang-Ming University, Taipei, Taiwan; 2 Institute of Biopharmaceutical Sciences, National Yang-Ming University, Taipei, Taiwan; 3 Department of Pathology and Laboratory Medicine, Taipei Veterans General Hospital, Taipei, Taiwan; 4 Division of Thoracic Surgery, Department of Surgery, Veterans General Hospital, Taipei, Taiwan; 5 Institute of Medicine, Chung-Shan Medical University, Taichung, Taiwan; 6 Institute of Molecular and Genomic Medicine, National Health Research Institutes, Zhunan, Taiwan; 7 Institute of Clinical Medicine, Medical College, National Cheng Kung University, Tainan, Taiwan; 8 Department of Life Science, Fu-Jen Catholic University, Taipei, Taiwan; University of Central Florida, United States of America

## Abstract

The epidermal growth factor receptor (EGFR), which is up-regulated in lung cancer, involves the activation of mitogenic signals and triggers multiple signaling cascades. To dissect these EGFR cascades, we used 14 different phospho-EGFR antibodies to quantify protein phosphorylation using an *in situ* proximity ligation assay (*in situ* PLA). Phosphorylation at EGFR-Thr654 and -Ser1046 was EGF-dependent in the wild-type (WT) receptor but EGF-independent in a cell line carrying the EGFR-L858R mutation. Using a ProtoAarray™ containing ∼5000 recombinant proteins on the protein chip, we found that AURKA interacted with the EGFR-L861Q mutant. Moreover, overexpression of EGFR could form a complex with AURKA, and the inhibitors of AURKA and EGFR decreased EGFR-Thr654 and -Ser1046 phosphorylation. Immunohistochemical staining of stage I lung adenocarcinoma tissues demonstrated a positive correlation between AURKA expression and phosphorylation of EGFR at Thr654 and Ser1046 in *EGFR*-mutant specimens, but not in *EGFR*-WT specimens. The interplay between EGFR and AURKA provides an explanation for the difference in EGF dependency between *EGFR*-WT and *EGFR*-mutant cells and may provide a new therapeutic strategy for lung cancer patients carrying *EGFR* mutations.

## Introduction

Lung cancer is the most common cause of cancer deaths worldwide, and the five-year relative survival rate of lung cancer patients is less than 15% [1]. There are two main types of lung cancers: small-cell lung cancer (SCLC, approximately 20% of lung cancers) and non-small-cell lung cancers (NSCLC, approximately 80% of lung cancers) [2], [3]. Epidermal growth factor receptor (EGFR), which is a receptor tyrosine kinase (RTK), initiates multiple signaling pathways related to cancer progression, such as those involved in cell proliferation, migration/invasion and the cell cycle [4]–[7]. Overexpression of EGFR is observed in approximately 50% of NSCLCs and is also associated with poor prognosis and a more aggressive disease course [8], [9]. *EGFR* mutations are frequently detected in NSCLC patients (10–40%) [10], [11]. Approximately 50% of *EGFR* mutations consist of deletions in exon 19, whereas 35–45% consist of the L858R mutation and 5% consist of insertions in exon 20 or the L861Q mutation [10]–[12]. Gefitinib (Iressa) and Erlotinib (Tarceva) are EGFR inhibitors that are used clinically for the treatment of advanced NSCLC, primarily that with *EGFR* mutations in the tyrosine kinase domains [13]–[16].

EGFR is activated by the binding of its cognate ligands, such as EGF and TGFα. Ligand binding to wild-type (WT) EGFR results in receptor dimerization and activation of the intrinsic kinase domain, followed by phosphorylation of specific tyrosine residues on the cytoplasmic tail [17]–[19]. The dysregulation of EGFR-activated pathways may result from mutations that cause ligand-independent receptor dimerization, activation and downstream signaling [16], [20]. Upon EGF stimulation, EGFR tyrosine phosphorylation is an “early event”, whereas EGFR serine/threonine phosphorylation, e.g. Ser967, occurs with a time delay [21], [22]. The phosphorylation of EGFR at many tyrosine sites after ligand stimulation initiates downstream signaling cascades, and the phosphorylation of EGFR at serine/threonine has been reported to attenuate these signals through negative feedback [23]–[25]. Many serine and threonine phosphorylation sites are present in EGFR, but their function remains unclear. Moreover, the signaling outcome induced by the phosphorylation of different sites on EGFR is complicated and remains to be elucidated for the development of therapeutic applications.

The AURKA protein kinase has attracted attention because its overexpression has been found in various epithelial malignant tumors [26], [27], such as breast [28], colon [29], ovarian [30] and lung cancers [31], as the result of gene amplification, transcriptional deregulation or defects in protein stability and the control of kinase activity [32]. Dysregulation of AURKA and EGFR is observed in different types of cancer and is an important indicator of prognosis in cancer development [33]. A previous study demonstrated that EGF-induced recruitment of nuclear EGFR and STAT5 to the AURKA promoter further increased AURKA gene expression [34]. Moreover, EGFR increases the protein expression of AURKA by activating the translational machinery via the ERK and AKT pathways [35]. These findings raise the possibility that these two proteins are functionally linked.

Recently, the *in situ* proximity ligation assay (*in situ* PLA) was developed to detect and visualize endogenous PPIs and post-translational modifications of proteins, e.g. phosphorylation, with high sensitivity and specificity [36], [37]. To detect protein phosphorylation, dual targets of primary antibody pairs [one that recognizes the target protein (e.g. EGFR) and another that recognizes the phospho-site of the target (e.g. pEGFR-Tyr1068)] were selected. If the targets of an antibody pair are in close proximity, secondary antibodies conjugated with oligonucleotides will be sufficiently close to serve as templates for the ligation of two additional linear oligonucleotides into a DNA circle. The DNA circle can be amplified with the oligonucleotide of one of the secondary antibodies using rolling circle amplification (RCA). The RCA product can then be hybridized with fluorescent-labeled oligonucleotides to generate a dot signal that indicates the subcellular location and frequency of phosphorylation [36], [37]. This technique has high specificity and sensitivity for the evaluation of protein phosphorylation and provides new opportunities to accurately quantify protein phosphorylation and signal transduction in cells.

Here, we used 14 different EGFR phosphorylation-site-targeted antibodies with *in situ* PLA to elucidate differences between EGFR-WT and EGFR-L858R mutant in lung cancer cells. Of particular interest is the identification of two EGF-independent phosphorylation sites (EGFR-Thr654 and EGFR-Ser1046) in cells carrying the EGFR-L858R mutation. Moreover, both EGFR-WT and EGFR-L858R mutant showed physical interactions with AURKA under EGF stimulation. EGFR-Thr654 and EGFR-Ser1046 phosphorylation decreased upon application of an AURKA inhibitor, but only in EGFR-L858R mutant cell lines, which raised the possibility of the therapeutic application of AURKA inhibitors in lung cancer patients.

## Results

### Profiling of EGFR phosphorylation in lung cancer cells

**Figure 1:**
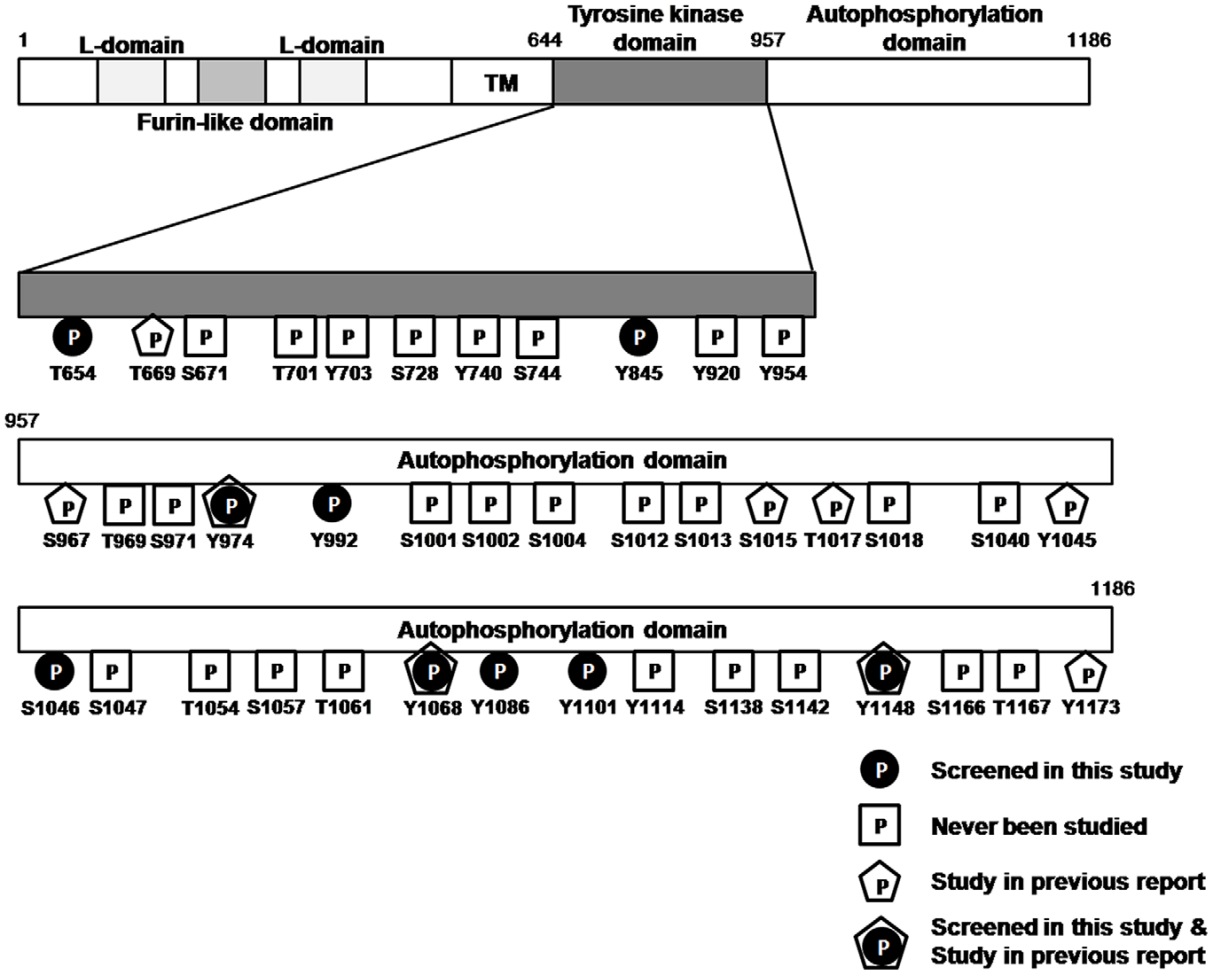
Schematic illustration of EGFR phosphorylation sites in tyrosine kinase domains and autophosphorylation domains. The EGFR phosphorylation site information was obtained from PubMed and PhosphoSitePlus (http://www.phosphosite.org).

**Figure 2:**
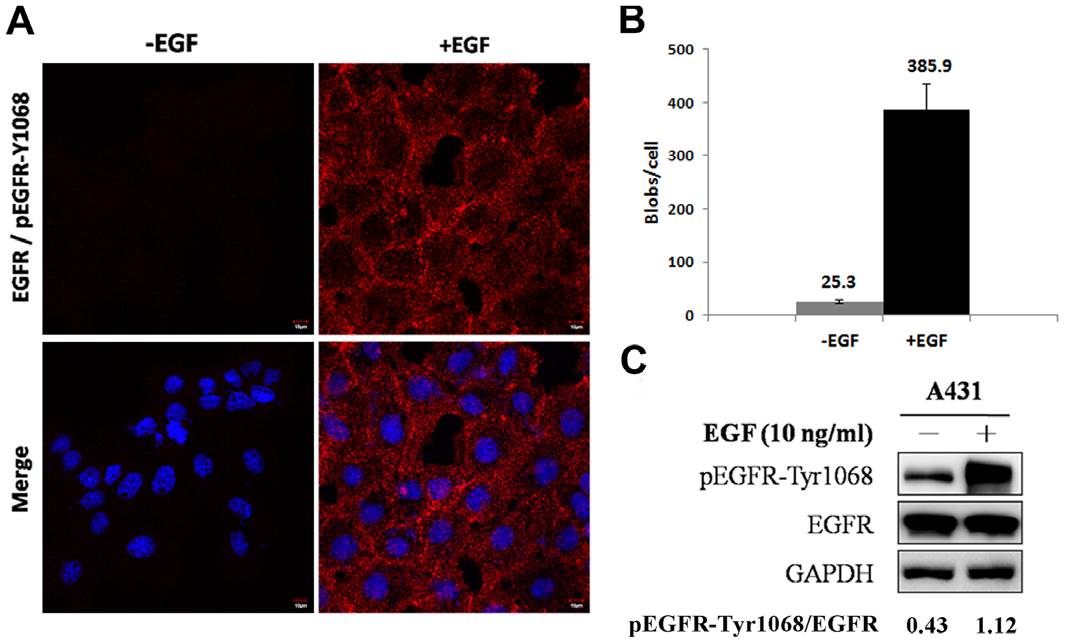
Detection of pEGFR-Tyr1068 upon EGF stimulation with i*n situ* PLA to confirm the specificity and sensitivity of *in situ* PLA. A431 cells were serum-starved for 16 hours and then treated with EGF (10 ng/ml) in serum-free medium for 10 minutes. (A) *In situ* PLA is highly sensitive for detection of the phosphorylation of pEGFR-Tyr1068 after EGF stimulation. (B) The quantification of these two signals is shown. (C) EGFR and pEGFR-Tyr1068 were detected in A431 cells by immunoblotting analysis.

EGFR phosphorylation plays a key role in modulating the activation of signaling pathways related to cancer progression [4]–[7]. There are numerous EGFR phosphorylation sites (http://www.phosphosite.org) [38], and many of them have not been studied extensively (Figure 1), because of a lack of antibodies and methods of detection. In the present study, 14 phosphorylated EGFR antibodies were used for *in situ* PLA to quantify the basal level of EGFR signaling in HeLa cells, which is the common cell line for screening (Table 1). All of the antibodies tested negative in the *in situ* PLA assay in HeLa cells, which is consistent with the idea that EGFR is activated upon the binding of its ligand and triggers downstream effectors through receptor dimerization and phosphorylation of specific tyrosine residues in the cytoplasmic tail [17]–[19]. To confirm the sensitivity and specificity of the *in situ* PLA, we then used A431 cells, which are well-known to express high levels of EGFR and hyper-phosphorylated EGFR-Tyr1068 under EGF stimulation. (Figure 2, the representative BlobFinder overlay images were shown in the Figure S1). Thus, A431 cells were chosen as a positive control. The pEGFR-Tyr1068 was upregulated by an approximately 15-fold increase upon EGF stimulation as determined by *in situ* PLA assay (Figure 2A and 2B), whereas it was only upregulated by 2.6-fold as determined by immunoblotting (Figure 2C), which indicates the high sensitivity of the *in situ* PLA assay.

**Table 1:** Fourteen EGFR tyrosine, serine and threonine phosphorylation sites were screened via *in situ* PLA and immunoblotting analyses.

EGFR phosphorylation site	Positive or negative of *in situ* PLA signal in HeLa cells (Blobs/cell)^a^	Positive or negative of immunoblotting signal with EGF stimulation in H1299 stable clones^b^	EGFR-WT cells^c^	EGFR-L858R cells^c^
EGFR-Thr654	Negative (17.8)	Positive	EGF-dependent	EGF-independent
EGFR-Thr669	Negative (9.8)	Negative	N/D	N/D
EGFR-Ser671	Negative (3.7)	Negative	N/D	N/D
EGFR-Ser744	Negative (3.3)	Negative	N/D	N/D
EGFR-Tyr845	Negative (17.2)	Positive	EGF-dependent	EGF-independent
EGFR-Tyr974	Negative (10.9)	Positive	EGF-dependent	EGF-independent
EGFR-Tyr992	Negative (0.7)	Positive	EGF-dependent	EGF-dependent
EGFR-Tyr1045	Negative (2.1)	Negative	N/D	N/D
EGFR-Ser1046	Negative (1.7)	Positive	EGF-dependent	EGF-independent
EGFR-Tyr1068	Negative (10.6)	Positive	EGF-dependent	EGF-dependent
EGFR-Tyr1086	Negative (20.8)	Positive	EGF-dependent	EGF-independent
EGFR-Tyr1101	Negative (8.8)	Positive	EGF-dependent	EGF-independent
EGFR-Tyr1148	Negative (19.9)	Positive	EGF-dependent	EGF-dependent
EGFR-Ser1166	Negative (0.8)	Negative	N/D	N/D

a : The criteria of positive *in situ* PLA signal was described in Method.

b : The negative signal of immunoblotting means the results with no signal or multiple bands.

c : N/D: not determined.

### Differential expression of EGFR phosphorylation after EGF stimulation between EGFR-WT and EGFR-mutant cells

**Figure 3:**
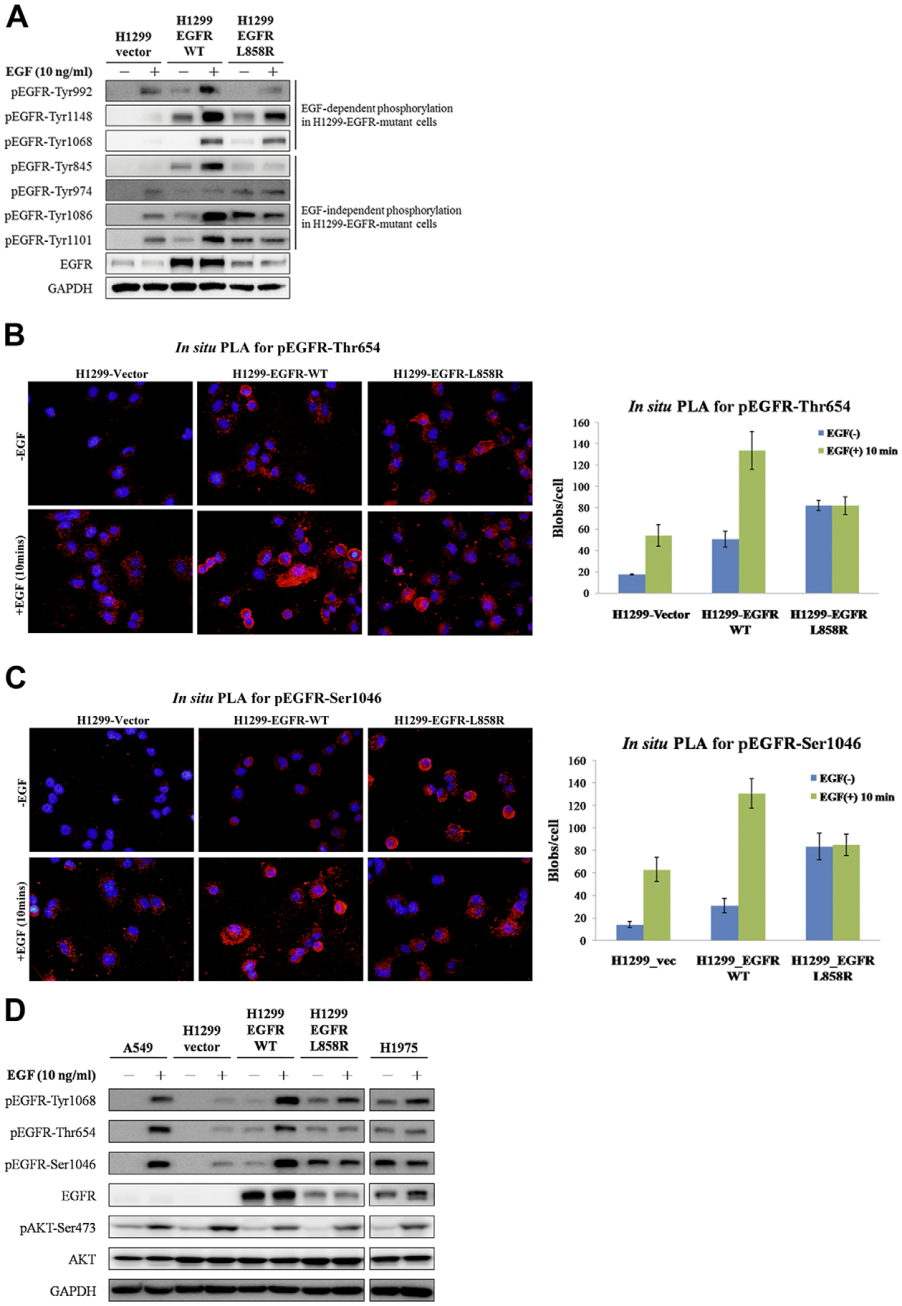
The EGFR phosphorylation sites showed distinct phosphorylation patterns after EGF stimulation in EGFR-WT and EGFR-mutant cells. H1299 cells stably expressed the empty vector, EGFR-WT or mutant EGFR-L858R. (A) After stimulation with EGF (10 ng/ml) in serum-free medium for 10 minutes, cell extracts were subjected to immunoblotting analysis with the indicated phospho-tyrosine antibodies. EGF-dependent (EGFR-Tyr992, -Tyr1148 and -Tyr1068) and EGF-independent [EGFR-Tyr845, -Tyr974, -Tyr1086 and -Tyr1101, -Thr654 (see below) and -Ser1046 (see below)] phosphorylation were observed in H1299 cells expressing EGFR-L858R mutant. The phosphorylation of EGFR-Thr654 (B) and -Ser1046 (C) with or without EGF treatment in different lung cancer cell lines was monitored via *in situ* PLA. The quantification of *in situ* PLA is shown on the right. EGFR-Thr654 and -Ser1046 were phosphorylated upon EGF treatment in H1299-EGFR-WT cells, whereas their phosphorylation was EGF-independent in H1299-EGFR-L858R cells. (D) Immunoblotting analysis of EGFR-Thr654 and -Ser1046 was performed in EGFR mutant cells (H1975, which contains the EGFR-L858R and -T790M mutants) and cells expressing EGFR-WT (A549). The phosphorylation of EGFR-Tyr1068 was induced by EGF. The phosphorylation of EGFR-Thr654 and -Ser1046 was EGF-independent in H1975 and H1299-EGFR-L858R cells as determined by immunoblotting.

EGFR activation initiates multiple signaling pathways, and the dysregulation of EGFR-activated pathways may be a consequence of various *EGFR* mutation [39]. To determine the differential phosphorylation status of EGFR phosphorylation sites, we detected EGFR phosphorylation at different sites in H1299 lung cancer cells that stably expressed an empty vector, EGFR-WT or EGFR-L858R mutant with or without ligand stimulation. In H1299-EGFR-WT stable cells, 9 of 14 EGFR phosphorylation sites were phosphorylated upon EGF stimulation. The rest of the pEGFR antibodies failed to show a signal on immunoblotting. In contrast, in the EGFR-L858R mutant cells, EGF-induced (EGFR-Tyr992, EGFR-Tyr1068 and EGFR-Tyr1148) and EGF-independent phosphorylation (EGFR-Thr654, EGFR-Tyr845, EGFR-Tyr974, EGFR-Ser1046, EGFR-Tyr1086, and EGFR-Tyr1101) were identified (Figure 3A–3C). Comparing the results of *in situ* PLA and immunoblotting, similar phosphorylation patterns were observed for all of the screened phosphorylation sites (Table S1 and Figure S2). The phosphorylation patterns of the 14 different EGFR phosphorylation sites as determined by *in situ* PLA and immunoblotting are summarized in Table 1.

### Phospho-EGFR-Thr654 and phospho-EGFR-Ser1046 exhibit EGF-independent phosphorylation in H1299-EGFR-L858R mutant cells

In the H1299-EGFR-L858R cell line, the phosphorylation of EGFR-Thr654 (Figure 3B) and EGFR-Ser1046 (Figure 3C) was identified as EGF-independent phosphorylation by *in situ* PLA and western blot analyses (the representative BlobFinder overlay images were shown in the Figure S3 and Figure S4). However, these two phosphorylation sites exhibited EGF-dependent phosphorylation in H1299 cells expressing EGFR-WT. Additionally, pEGFR-Thr654 and pEGFR-Ser1046, which were considered to be phosphorylated by other kinases [40]–[42] but not by EGFR auto-phosphorylation, were selected to verify the functional effects on EGFR signaling pathway.

We further evaluated whether the EGF-independent phosphorylation of EGFR-Thr654 and -Ser1046 only occurs in cells with overexpression of EGFR-L858R and is caused by dysregulation of the EGFR signaling pathway. We used A549 (lung cancer cells with endogenous EGFR-WT) and H1975 (lung cancer cells with endogenous mutated EGFR-L858R-T790M) to monitor them. Figure 3D shows that the phosphorylation of EGFR-Thr654 and -Ser1046 was EGF-independent in cells expressing exogenous and endogenous EGFR-L858R. Thus, different EGFR serine/threonine phosphorylation patterns were observed as EGF-independent phosphorylation in EGFR-L858R mutant cells.

### The physical interaction between AURKA and EGFR

**Figure 4:**
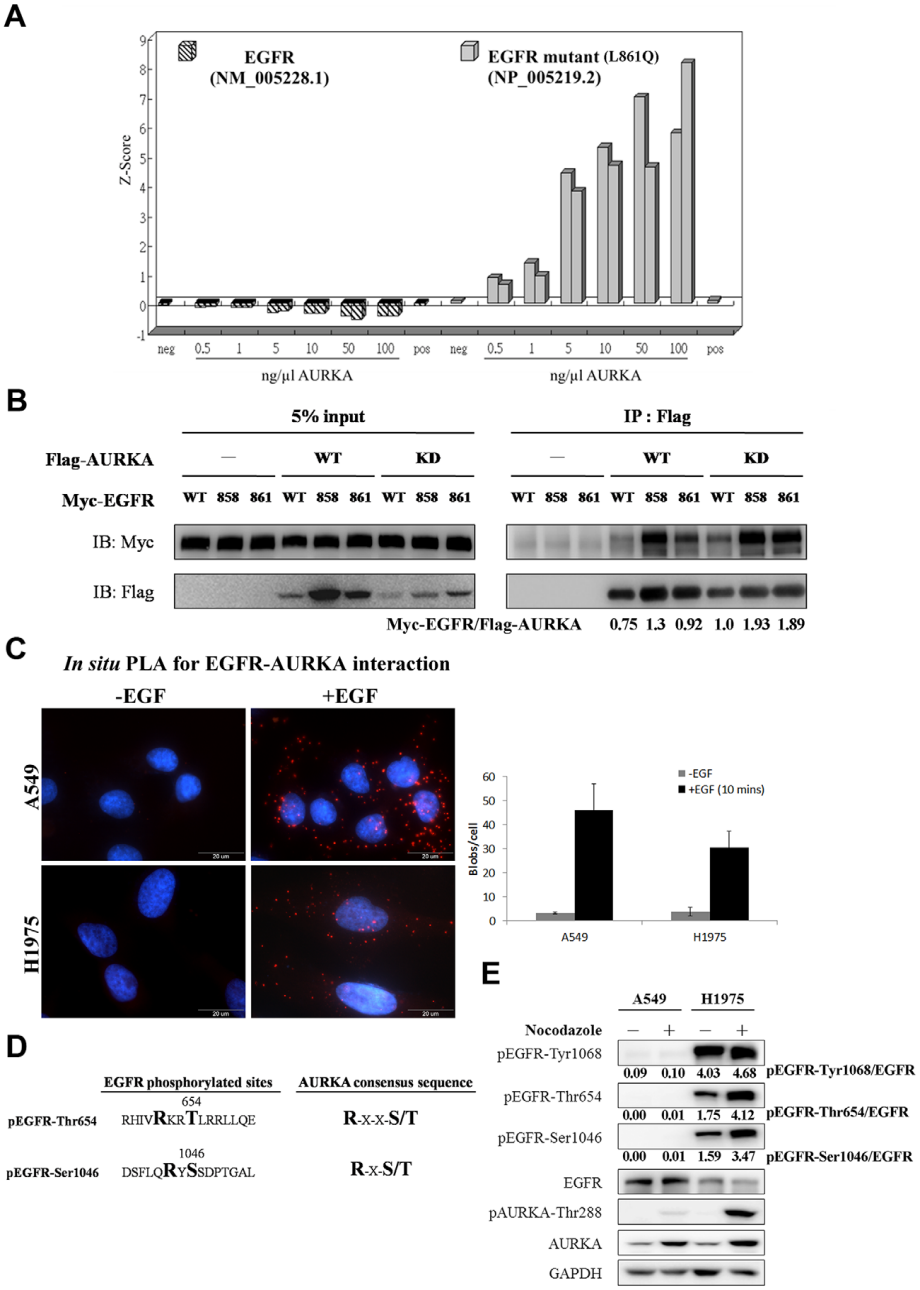
Characterization of interaction between AURKA and EGFR. (A) We used ProtoArray™ to identify EGFR as a novel interaction partner for AURKA. Briefly, expressed, recombinant his-tagged human AURKA was probed on ProtoArray™ human protein microarrays at seven concentrations (0, 0.5, 1.0, 5.0, 10, 50 and 100 ng/µl) in duplicate. It should be noted that EGFR-L861Q, but not EGFR-WT, was initially identified to interact with AURKA. (B) HEK293T were co-transfected with Myc-EGFR (EGFR-WT, EGFR-L858R and -L861Q mutants) and Flag-AURKA [AURKA-WT and AURKA-kinase dead (KD)]. After transfection for 48 hours, the cell extracts were subjected to co-immunoprecipitation with an anti-FLAG M2 affinity gel. Protein expression was determined by immunoblotting with anti-Myc and anti-FLAG antibodies. It showed that both EGFR-WT and mutated EGFR associates with AURKA-WT and AURKA-KD. Similar results were observed in at least three independent experiments. (C) The EGFR-AURKA interaction with or without EGF stimulation (10 ng/ml for 10 minutes) was determined via *in situ* PLA in A549 (EGFR-WT) and H1975 (EGFR-L858R-T790M mutations). Both EGFR-WT and -L858R mutant were associated with AURKA under EGF stimulation. The quantification of *in situ* PLA signals was shown on the right. (D) The phosphorylation sites of EGFR-Thr654 and -Ser1046 matched the substrate consensus sequences for AURKA. (E) H1975 cells showed more obvious phosphorylation at EGFR-Thr654, EGFR-Ser1046 and AURKA-Thr288 in nocodazole-arrested M-phase than in interphase.

AURKA is a kinase that is highly expressed in mitosis and has been implicated in the cell transformation processes [43]. In a search for additional substrates and interacting proteins using a ProtoAarray™ [44] that contained ∼5000 recombinant proteins on the protein chip (www.invitrogen.com/protoarray), we found that AURKA interacted with the EGFR-L861Q mutant, which comprises ∼5% of *EGFR* mutations in lung cancer patients [10], but not EGFR-WT (Figure 4A). To confirm this interaction, which might be regulated by the kinase activity of AURKA, AURKA-WT and AURKA-KD (AURKA-K162I mutant, which is kinase-dead), were used for co-immunoprecipitation, which showed that EGFR-WT and EGFR-L858R interacted with AURKA-WT and AURKA-KD under the overexpression of EGFR and AURKA (Figure 4B). The results indicated that the catalytic activity of AURKA does not modulate the interaction with EGFR. Since EGFR-L858R mutant frequently (approximately 35–45%) appears in NSCLC patients [12], we chose H1299-EGFR-L858R stable cells to further characterize the interaction with AURKA. To examine whether the endogenous EGFR-AURKA interactions exist in lung cancer cells and whether it is EGF dependent, we applied *in situ* PLA to determine the EGFR-AURKA interaction in A549 and H1975 with or without EGF stimulation (Figure 4C). It showed both EGFR-WT and EGFR-L858R interacted with AURKA under EGF stimulation.

### EGFR-Thr654 and EGFR-Ser1046 match AURKA phosphorylation consensus sequences

Because AURKA associated with EGFR, we next investigated whether AURKA phosphorylates EGFR at Thr654 and Ser1046. The neighboring sequences for the EGFR phosphorylation sites at Thr654 and Ser1046 are RHIVRKRT^654^LRRLLQE and DSFLQRYS^1046^SDPTGAL, respectively. The AURKA phosphorylation motifs contain R-X-S/T, K-X-S/T, K-R-X-S/T, R-R-X-S/T and R-X-X-S/T (X indicates any amino acid) [45]. Based on these motifs, EGFR-Thr654 matched the R-X-X-S/T consensus sequence and EGFR-Ser1046 matched the R-X-S/T consensus sequence of AURKA (Figure 4D). Although EGFR-Thr654 was reported to be phosphorylated by protein kinase C (PKC) [42], PKC may not be the only kinase that phosphorylates EGFR at this residue. Because the kinase activity of AURKA increases in M-phase [46], we first characterized the phosphorylation of endogenous EGFR-Thr654 and EGFR-Ser1046 at M-phase in A549 and H1975 cells. H1975 cells demonstrated more obvious phosphorylation of EGFR-Thr654, EGFR-Ser1046 and AURKA-Thr288 than A549 cells. In contrast, EGFR-Tyr1068 demonstrated the same phosphorylation pattern in M-phase and interphase (Figure 4E), which suggests that the expression patterns of phosphorylated EGFR-Thr654 and EGFR-Ser1046 were the same with that of AURKA particularly in EGFR-mutant cells.

### EGFR-Thr654 and EGFR-Ser1046 phosphorylation occurs later than EGFR-Tyr1068 phosphorylation after EGF stimulation

**Figure 5:**
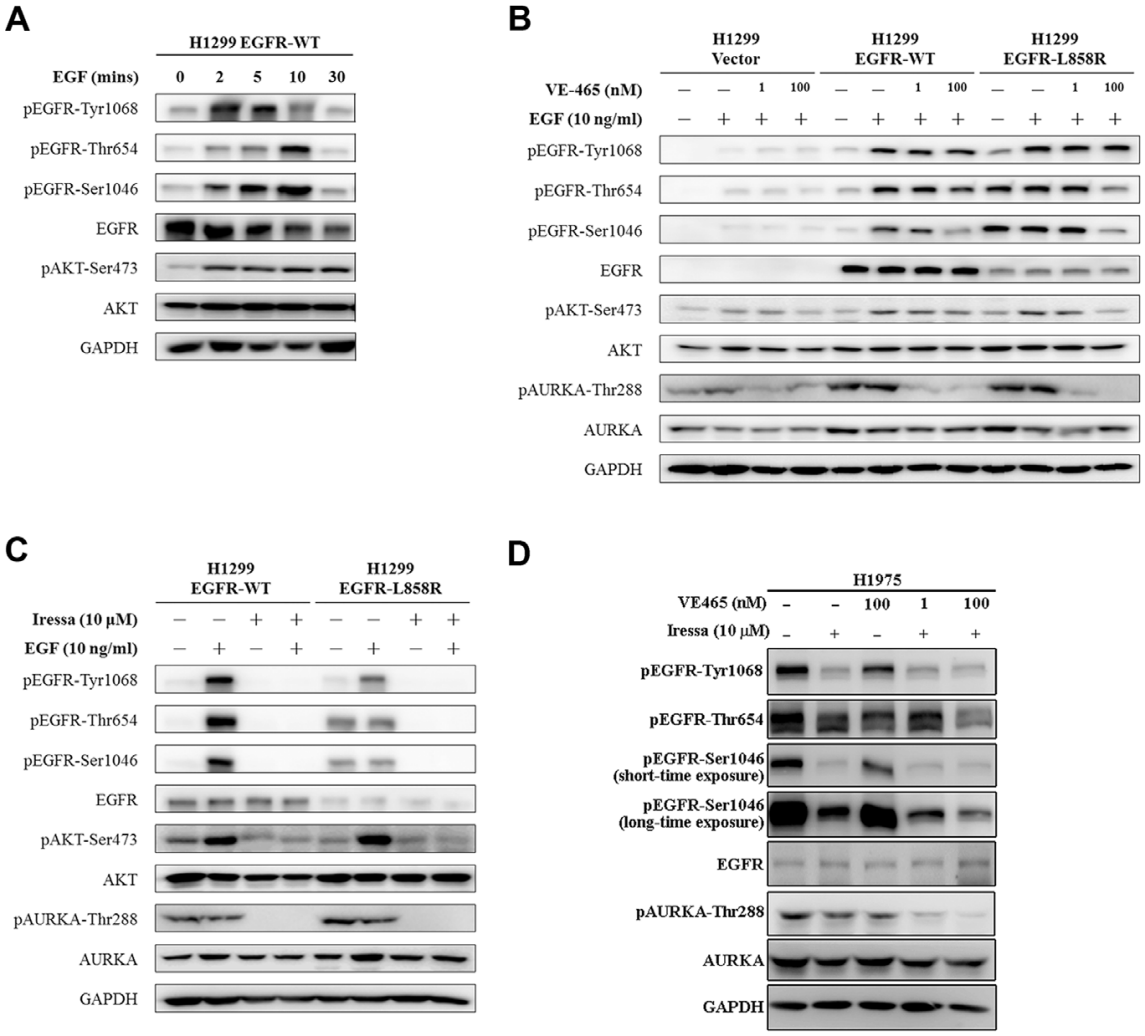
VE-465 and Iressa inhibit the phosphorylation of EGFR and AURKA phosphorylation. (A) The phosphorylation of EGFR-Tyr1068 was faster than that of EGFR-Thr654 and EGFR-Ser1046 under the EGF (10 ng/ml) stimulation. (B) The cells were serum-starved in the presence of VE-465 (1 nM or 100 nM), which is an AURKA inhibitor, for 16 hours and then treated with EGF (10 ng/ml) for 10 minutes. The phosphorylation of EGFR-Thr654 and EGFR-Ser1046, but not pEGFR-Tyr1068, was suppressed by VE-465. (C) The cells were serum-starved in the presence of Iressa (10 µM) for 16 hours and then treated with EGF (10 ng/ml) for 10 minutes. Iressa, which is an EGFR inhibitor, was used to monitor EGFR signaling in H1299 cells stably expressing EGFR-WT and EGFR-L858R mutant. The phosphorylation by AURKA at Thr288 was suppressed by Iressa in both cell lines. (D) H1975 cells were treated with VE-465 and/or Iressa (10 µM) for 16 hrs. VE-465 and Iressa can suppress pEGFR-Thr654 and -Ser1046 in H1975.

The phosphorylation of EGFR-Tyr1068 after EGF stimulation observed at 0, 2, 5, 10 and 30 minutes in H1299 cells expressing EGFR-WT was faster than the phosphorylation of Thr654 and Ser1046. The phosphorylation of EGFR-Tyr1068 peaked at 2 minutes after EGF stimulation, but EGFR phosphorylation at Thr654 and Ser1046 peaked at 10 minutes after EGF stimulation (Figure 5A). In contrast, in EGFR-L858R cells, there was no difference in phosphorylation kinetics between Thr654 and Ser1046 (Figure S5). Thus, EGFR tyrosine phosphorylation occurs prior to other serine/threonine phosphorylation at Thr654 and Ser1046, which consisted with the previous study of EGFR serine/threonine phosphorylation with time delay [21], [22].

### AURKA inhibitor suppresses EGFR serine/threonine phosphorylation

To investigate the relationship between EGFR serine/threonine phosphorylation and AURKA, an AURKA inhibitor (VE-465), which suppresses AURKA activity as measured by Thr288 phosphorylation [47] but not the protein level of AURKA, was applied to monitor the phosphorylation of EGFR-Thr654 and EGFR-Ser1046 as well as the EGFR signaling pathway (pAKT-Ser473). Under VE-465 treatment, the phosphorylation of EGFR-Thr654 and -Ser1046 decreased, but EGFR-Tyr1068 phosphorylation was not affected (Figure 5B), which indicates that the phosphorylation of EGFR-Thr654 and EGFR-Ser1046 was regulated by AURKA kinase activity.

### Iressa is an EGFR inhibitor that suppresses the AKT pathway and inhibits AURKA phosphorylation

To evaluate the regulation of EGFR and AURKA in the EGFR signaling pathway, an EGFR inhibitor (Iressa) was used to block EGFR autophosphorylation and its downstream signaling. Iressa suppressed AURKA activity and the phosphorylation of EGFR-Thr654 and EGFR-Ser1046 (Figure 5C), which suggests that AURKA may serve as a downstream target of EGFR. In addition to the EGFR-overexpressed cells, we also showed the phosphorylation status of EGFR-Thr654 and -Ser1046 in endogenous L858R mutant cells (H1975) under treatment of VE-465 and Iressa (Figure 5D). Decrease of the pEGFR-Thr654 and -Ser1046 was observed in H1975 with VE-465 treatment. Although Iressa inhibited pEGFR-Thr654 and -Ser1046 in H1975, the combination of Iressa and VE-465 may further decrease pEGFR-Thr654 and -Ser1046 in H1975, implying that these two pEGFR sites were regulated by both EGFR and AURKA kinase activity.

### IHC analysis of AURKA, pEGFR-Thr654 and pEGFR-Ser1046 in stage I lung adenocarcinoma

**Figure 6:**
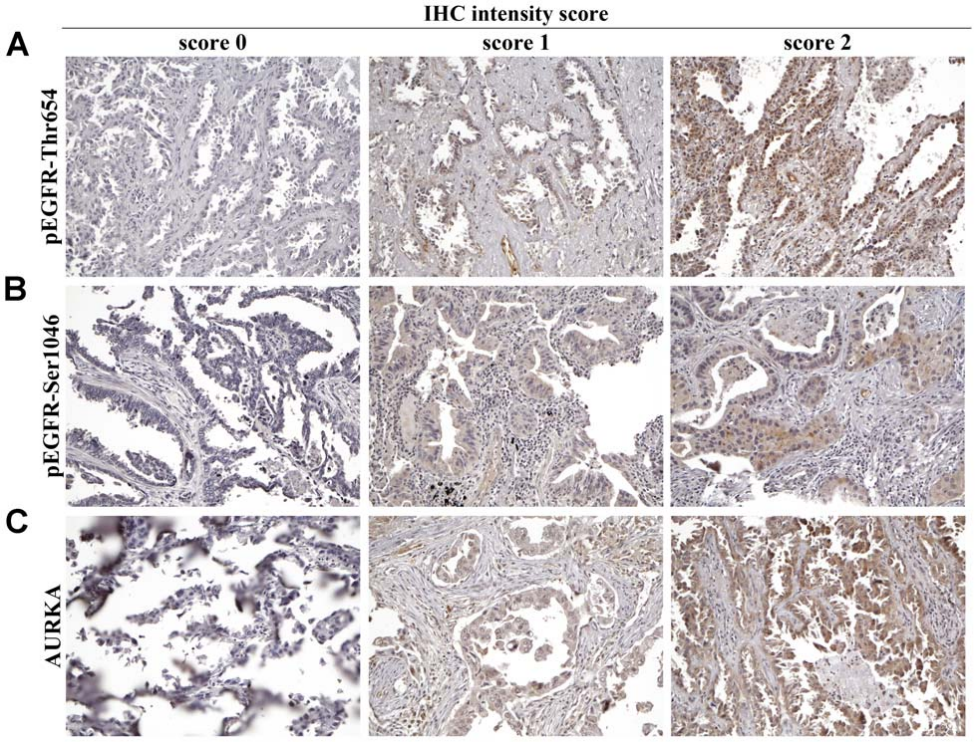
The representative expression of the IHC intensity score for AURKA, pEGFR-Thr654 and pEGFR-Ser1046 in stage I lung adenocarcinoma patients with *EGFR* mutations. Each column indicates a patient and each row indicates an antibody. The IHC intensity in the patients was defined as 0, 1 and 2 for AURKA, pEGFR-Thr654 and pEGFR-Ser1046 staining.

To further evaluate the relationship between AURKA and EGFR phosphorylation, 25 stage I lung adenocarcinoma specimens were subjected to IHC staining of pEGFR-Thr654, pEGFR-Ser1046 and AURKA (Figure 6). Eighteen of these NSCLC samples had *EGFR* mutations as determined by sequence analysis of the *EGFR* tyrosine kinase domains. The expression of pEGFR-Ser1046 and AURKA was significantly higher in tumors than in the adjacent normal tissue (p = 0.001) of 25 NSCLC patients (Table 2). Moreover, the expression of AURKA showed a positive correlation with a significant correlation coefficient with pEGFR-Thr654 (p<0.05) and pEGFR-Ser1046 (p<0.001) in the patients with *EGFR* mutant, but not with *EGFR*-WT, as analyzed by the Spearman's non-parametric correlation test (Table 3). In summary, this immunochemical staining provides evidence of a possible link between AURKA and mutated EGFR via -Thr654 and -Ser1046.

**Table 2:** Wilcoxon two-sample t-test for IHC staining in 25 NSCLC patients.

	P-value	
Antibody	Normal vs. Tumor	WT vs. Mutant^a^
pEGFR-Thr654	0.184	0.141
pEGFR-Ser1046	0.001*	0.180
AURKA	0.001*	0.414

a : In total, 7 EGFR-WT and 18 mutant EGFR (Mutant) samples were examined in this study. The IHC staining was compared between WT and mutant samples.

**Table 3:** Spearman's non-parametric correlation test for IHC staining of 25 NSCLC patients.

	Correlation coefficient		
	25 patients	7 EGFR-WT patients	18 EGFR mutant patients
AURKA vs. pEGFR-Thr654	0.294*	0.005	0.366*
AURKA vs. pEGFR-Ser1046	0.372**	-0.108	0.534**

* : p<0.05;

** : p<0.01.

In conclusion, the interaction between AURKA and the EGFR-L858R mutant may explain the difference in signaling pathways between *EGFR*-WT and mutant cells, which are frequently observed in NSCLC patients, and may thus provide valuable information for therapeutic applications.

## Discussion

The dysregulation of EGFR-activated pathways is often associated with receptor mutations and predicts the response to tyrosine kinase inhibitors in NSCLC patients [48], [49]. The profile of EGFR phosphorylation in *EGFR* mutant lung cancer cells remains unclear. In this study, we (1) systematically analyzed endogenous EGFR phosphorylation changes to identify distinct EGF dependency in different *EGFR* genotypes in lung cancer cells; (2) demonstrated a novel relationship between AURKA and EGFR, which might be mediated via an AURKA-elicited phosphorylation event at EGFR-Thr654 and -Ser1046 in lung cancer cells; and (3) determined the expression of AURKA, EGFR-Thr654 and EGFR-Ser1046 in lung adenocarcinoma tissues. These data highlight the need for further analysis for the relationship between EGFR and AURKA in *EGFR*-mutant patients and provides new insight into the therapeutic application of AURKA inhibitors in lung cancer patients.

EGFR-Thr654 is known to be phosphorylated by PKC and is involved in negative regulation of EGFR signaling after EGF stimulation [50], [51]. Moreover, the phosphorylation of EGFR at residue Thr654 was reported to be associated with radiation-induced nuclear EGFR translocation, which promotes cell proliferation and metastasis by increasing the transcription of cyclin D1, iNOS and B-Myb [52]–[54]. After the cell is subjected to ionizing radiation, the DNA-dependent kinase (DNA-PK) interacts with nuclear EGFR and repairs non-homologous end-joining DNA-double strand breaks, which causes radioresistance in radiotherapy. In the present study, EGFR-Thr654 was constitutively phosphorylated in EGFR-L858R mutant cells. It would be of interest to examine whether EGFR-Thr654 phosphorylation affects radioresistance during radiotherapy in the EGFR-L858R mutant cells. Moreover, we also demonstrated that an AURKA inhibitor decreased the phosphorylation of EGFR-Thr654, which might provide a potential avenue for a new clinical strategy with an AURKA inhibitor.

Cellular stressors, such as TNF-α and UV irradiation, have been reported to induce the phosphorylation of EGFR-Ser1046 via p38 MAPK and then cause EGFR desensitization upon EGF stimulation and endocytosis [55], [56]. In ligand-induced EGFR activation, several tyrosine residues, e.g. Tyr-1045 and Tyr-1068, are primarily phosphorylated. Grb2 then binds to phosphorylated tyrosine to activate MAPKs. Subsequently, the MAPKs phosphorylate serine/threonine residues, such as Ser-1046, on EGFR as a feedback control. The phosphorylation of EGFR-Ser1046 by p38 was also recently determined to be a key signal that prevents the pro-apoptotic cleavage of caspase and PARP upon TNFα stimulation [57]. Upon EGF stimulation, EGFR is only slightly phosphorylated at Ser1046 to provide an anti-apoptosis effect [57]. In this study, we demonstrated that EGFR-Ser1046 phosphorylation is EGF-independent in EGFR-L858R mutant cells, whereas EGFR-Ser1046 phosphorylation is EGF-dependent in EGFR-WT cells. Thus, whether EGFR-Ser1046 phosphorylation in EGFR-L858R mutant cells results in an anti-apoptotic effect warrants further investigation.

H1299-EGFR-L858R cells have a lower EGFR level than that in the H1299-EGFR-WT cells; however, EGF-independent basal phosphorylation levels in L858R cells on multiple Ser, Thr, and Tyr residues are stronger than those in EGFR-WT cells (Figure 3). This indicates that while the L858R mutant receptor showed a lower expression level, it was hyper-phosphorylated than the wild-type receptor. Moreover, EGFR L858R mutant is known to be more active than the EGFR-WT in previous study [16]. Overall, the EGFR phosphorylation in L858R cells was less responsive to EGF. This could be due to the higher basal phosphorylation levels of the mutant receptor or due to its lower expression level. Because EGFR signaling can increase the protein expression of AURKA [34], [35], AURKA may also regulate EGFR phosphorylation. Here, we demonstrated that the EGFR-AURKA interaction is only occurred after EGF stimulation (Figure 4C), implying that AURKA might participate in EGFR signaling cascade. In nocodazole-arrested M-phase, EGFR-mutant cells showed more obvious phosphorylation on EGFR-Thr654, EGFR-Ser1046 and AURKA-Thr288 than cells expressing EGFR-WT (Figure 4E). EGFR activation occurs through the AKT pathway and EGFR can upregulate AURKA expression by activating the translational machinery via the AKT pathway [35]. The AURKA inhibitor VE-465 suppressed the AKT pathway and inhibited EGFR serine/threonine phosphorylation, especially in cells carrying *EGFR* mutations, but did not affect EGFR tyrosine phosphorylation (Figure 5B). When the cells were treated with the EGFR inhibitor Iressa to block EGFR autophosphorylation, the phosphorylation of AURKA vanished (Figure 5C), which suggests that EGFR mutants may constitutively activate phosphorylation and enhance downstream signaling to promote carcinogenesis. However, the relationship of AURKA, EGFR-WT, and mutant EGFR must be further evaluated to identify therapeutic targets for lung cancer patients.

In summary, by profiling EGFR phosphorylation in wild-type and mutant lung cancer cells, we identified a relationship between *EGFR* mutations and the phosphorylation of EGFR-Thr654 and -Ser1046, which was associated with AURKA expression in lung cancer patients and may be elicited by AURKA. The interaction between EGFR and AURKA in *EGFR* mutation cells suggests that AURKA inhibitors may have therapeutic impact. We anticipate that this study will facilitate the development of an effective therapeutic strategy for lung cancer.

## Materials and Methods

### Cell lines, cell cycle synchronization, and EGF stimulation

The establishment of H1299 clones stably expressing an empty vector (H1299-Vec), EGFR-WT (H1299-EGFR-WT) or EGFR-L858R mutant (H1299-EGFR-L858R) was described previously [16]. A549 (with EGFR-WT), H1975 (with endogenous mutated EGFR-L858R-T790M) and H1299 stable clones were cultured in RPMI-1640 medium, and A431 cells were maintained in DMEM supplemented with 10% fatal bovine serum (FBS), 100 U penicillin, 0.1 mg/ml streptomycin, and 2 mM L-glutamine. All cell culture reagents were from Invitrogen. To synchronize the cell cycle of A549 and H1975 lung cancer cell lines, exponentially growing cells were incubated with 100 ng/ml nocodazole (Sigma) for 16 hours. To stimulate the cells with EGF, the cells were serum-starved for 16 hours, followed by a 10-minute treatment with EGF (10 ng/ml) in serum-free medium.

### Immunoblotting analysis

The cell lysates (50 µg) were subjected to SDS-polyacrylamide gel electrophoresis (SDS-PAGE) and subsequently transferred to polyvinylidenedifluoride (PVDF) membrane (Millipore) using the Bio-Rad transfer system. The PVDF membrane was blocked with 5% non-fat skim milk (BD Bioscience) at room temperature for 1 hour, followed by incubation with the primary antibody at 4°C overnight. All phospho-polyclonal EGFR antibodies (pEGFR-Thr654, pEGFR-Thr669, pEGFR-Ser671, pEGFR-Ser774, pEGFR-Tyr845, pEGFR-Tyr974, pEGFR-Tyr992, pEGFR-Tyr1045, pEGFR-Ser1046, pEGFR-Tyr1086, pEGFR-Tyr1101, pEGFR-Tyr1148, and pEGFR-Ser1166) were from Abnova Corporation. The monoclonal antibodies used included those targeting pEGFR-Tyr1068 (Cell Signaling), EGFR (Cell Signaling), pAKT-Ser473 (Cell Signaling), pAURKA-Thr288 (Abcam), EGFR (Abcam), AKT (Cell Signaling) and AURKA (BD Bioscience). All the antibodies used in this study were listed in the Table S2. The membrane was washed with TBST buffer three times for 10 minutes each and then incubated with the secondary antibody, which was conjugated to horseradish peroxidase (Bio/Can Scientific), at room temperature for 2 hours. The signals of the secondary antibodies were visualized by adding chemiluminescent HRP substrate (Millipore) and detected using a Fujifilm LAS4000 luminescent image analysis system.

### *In situ* proximity ligation assay

The cells were stimulated with EGF (10 ng/ml) for 10 minutes and fixed in 3.7% paraformaldehyde for 3 minutes at room temperature. After washing with PBS, the cells were permeabilized with 0.2% TritonX-100 in PBS for 3 minutes at room temperature. To reduce the non-specific signals, the cells were incubated with a blocking solution (OLINK Bioscience) for 30 minutes at 37°C. Primary 1× antibody diluent (OLINK Bioscience) with two primary antibodies (1∶50 dilution for anti-EGFR mouse monoclonal antibodies and 1∶1200 for pEGFR rabbit polyclonal antibodies) was added to the cells and incubated overnight at 4°C. The negative control was performed by only one primary antibody (pEGFR rabbit polyclonal antibody) into cells for incubation at 4°C overnight. Then, the PLA Probe anti-Mouse PLUS and anti-Rabbit Minus (OLINK Bioscience), which were secondary antibody with specific oligonucleotides, were added for the incubation for 1 hour at 37°C. All of the procedures were performed according to the manufacturer's instructions. The images of the cells were acquired using an Olympus BX61 microscope (Olympus). Images for each slide with an *in situ* PLA sample were acquired at 5 different fields with 2 z-axis images. Then, the images were analyzed with BlobFinder V3.2 image analysis software, which automatically counts the number of spots per cell. The signal ratio of a dot signal was defined as 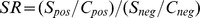. The signal of *in situ* PLA was determined to be positive only if 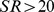 and 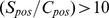, where 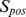 is the signal and 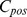 is the cell number for the dual-recognition with one EGFR antibody and one phospho-EGFR antibody; 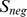 is the signal and C*_neg_* is the cell number for the negative control, in which only added one pEGFR rabbit polyclonal antibody. All the experiments were repeated at least 3 times except the one-shot screening of 14 EGFR phosphorylation status by *in situ* PLA (Table 1). All *in situ* PLA signals and their error bars were calculated from 3 different experiments. The antibodies used in this study were listed in the Table S2.

### Protein arrays

In this study, proteins that interact with AURKA were detected using a ProtoArray™ Human Protein Array (version 3.0) (Invitrogen) [44]. The 5,056 human proteins, which were expressed as N-terminal GST-tagged proteins in a baculovirus expression system, were spotted in duplicate on a glass slide (ProtoArray™). The recombinant His-tagged human AURKA was probed on the ProtoArray™ at seven concentrations (0, 0.5, 1.0, 5.0, 10, 50, and 100 ng/µl) in duplicate. All proteins that interacted with AURKA were evaluated by Z-score ranking and compared with the negative control.

### Transient transfection

HEK293T cells were co-transfected with Myc-EGFR [wild-type EGFR (WT), EGFR-L858R or -L861Q mutants] and Flag-AURKA [wild-type (WT) or kinase-dead (KD)] with serum-free medium by Lipofectamine® 2000 (Invitrogen) at 70% confluency. All procedure of transfection was according to the manufacturer's instructions. After cell transfection for 5 hours, the serum-free medium was changed to 10% FBS medium for the cells continuously growth. Then, cells were harvested after transfection for 48 hours.

### Immunoprecipitation

HEK293T cells were co-transfected with Myc-EGFR (EGFR-WT, EGFR-L858R or EGFR-L861Q mutants) and Flag-AURKA (AURKA-WT or AURKA-KD). After transfection for 48 hours, the cell extracts, containing 1 mg of total cellular protein, were supplemented with 1 µg of mouse IgG together with 20 µl protein A/G PLUS-Agarose (Santa Cruz) at 4°C for 1 hour to obtain pre-cleaned lysates. Subsequently, the pre-cleaned lysates were subjected to co-immunoprecipitation with the A/G PLUS-Agarose (Santa Cruz) and an anti-FLAG M2 (Sigma-Aldrich) or anti-Myc (Cell Signaling) antibody at 4°C overnight. The beads were recovered by centrifugation, washed three times with TBS buffer (50 mM Tris-HCl, 150 mM NaCl, and pH 7.4), resuspended in SDS sample buffer, heated at 95°C for 10 minutes, analyzed by SDS-PAGE and immunoblotted with anti-Myc (Cell Signaling) and anti-FLAG M2 (Sigma-Aldrich) monoclonal antibodies.

### Immunohistochemistry

Specimens from 2001 to 2007 for female NSCLC patients who had never smoked and who had stage I adenocarcinoma were collected from the surgical pathology archives at Taipei Veterans General Hospital. The study was approved (VGHIRB No.: 95-06-21A) by the Institutional Review Board of Taipei Veterans General Hospital on June 8, 2006 and was valid until June 7, 2009. The Institutional Review Board performs its functions according to written operating procedures and complies with GCP and with the applicable regulatory requirements. All patients gave informed consent and signed the consent form individually. Study samples, including tumor and adjacent normal tissues, were obtained from the diagnostic biopsy, and adjacent normal tissues were obtained from neighboring sites outside of the tumor. Sections for each tissue microarray (4 µm thickness) were placed in a 65°C oven for 15 minutes. The sections were then de-paraffinized in xylene twice for 5 minutes and rehydrated through an ethanol gradient to water (100%, 95%, 70% ethanol). Endogenous peroxidase activity was blocked by covering the tissue with a blocking solution for 30 minutes. Heat-induced epitope retrieval was performed in an antigen retrieval solution at 100°C for 10 minutes with a microwave and the sections were then incubated with a rabbit polyclonal anti-pEGFR-Thr654 antibody (1∶300 dilution, Abnova), rabbit polyclonal anti-pEGFR-Ser1046 antibody (1∶100 dilution, Abnova), and rabbit polyclonal anti-AURKA antibody (1∶400 dilution, Sigma-Aldrich). Finally, freshly prepared DAB substrate was added to the sections until suitable staining developed. The IHC results were assessed in a blinded fashion without knowledge of the patient's identity and clinical history. The extent and intensity of staining in the tumor cells were assessed using a semi-quantitative scale. The extent of staining in the tumors was scored as follows: 0 (0% immunoreactivity), 1 (≦10% immunoreactivity), 2 (11–50% immunoreactivity) and 3 (>50% immunoreactivity). The staining intensity was graded using the following scale: 0 (negative), 1 (weak), 2 (moderate) and 3 (strong). Finally, for each tumor, an overall score was calculated for the extent and intensity that ranged from 0 to 9.

## Supporting Information

Figure S1 **The calculation of** ***in situ*** **PLA signal in** **Figure 2A** **by BlobFinder.** The raw images (RGB images) of Figure 2 were converted into gray-scale images (the white dots were *in situ* PLA signals). The *in situ* PLA signals, which were calculated by BlobFinder, were outlined with red dots. The white square of the left image was enlarged in the right. (A) The *in situ* PLA signal of pEGFR-Tyr1068 in A431 cells without EGF stimulation. (B) The *in situ* PLA signal of pEGFR-Tyr1068 in A431 with EGF stimulation. Green: nuclear.
(TIF)

Figure S2 **The determination of phosphorylation status of 9 phospho-EGFR sites by** ***in situ*** **PLA in the H1299 cells with expression of vector control (H1299-Vector), wild-type EGFR (H1299-EGFR-WT) and EGFR with L858R mutant (H1299-EGFR-L858R).** The cells were stimulated with or without EGF (10 ng/ml) for 10 minutes followed by serum starvation for 16 hours. The quantification of *in situ* PLA signal was shown in the right.
(TIF)

Figure S3 **The calculation of** ***in situ*** **PLA signal of pEGFR-Thr654 in** **Figure 3B** **by BlobFinder.** The raw images (RGB images) of Figure 3B were converted into gray-scale images (the white dots were *in situ* PLA signals). The *in situ* PLA signals, which were calculated by BlobFinder, were outlined with red dots. (A) The *in situ* PLA signals of pEGFR-Thr654. (B) The enlarged images of (A). Green: nuclear.
(TIF)

Figure S4 **The calculation of** ***in situ*** **PLA signal pEGFR-Ser1046 in** **Figure 3C** **by BlobFinder.** The raw images (RGB images) of Figure 3C were converted into gray-scale images (The white dots were *in situ* PLA signals). The *in situ* PLA signals, which were calculated by BlobFinder, were outlined with red dots. (A) The *in situ* PLA signals of pEGFR-Ser1046. (B) The enlarged images of (A). Green: nuclear.
(TIF)

Figure S5 **The phosphorylation kinetics of EGFR-Tyr1068, EGFR-Thr654, and EGFR-Ser1046 under the EGF (10 ng/ml) stimulation in H1299-** ***EGFR*** **-L858R cells.** The phosphorylation kinetics of EGFR-Tyr1068, EGFR-Thr654, and EGFR-Ser1046 were not significantly changed when H1299-*EGFR*-L858R cells were treated with 10 ng/ml EGF for various time points.
(TIF)

Table S1 **The quantification of the phosphorylation status of 9 phospho-EGFR sites in the H1299 cells with expression of vector control (H1299-Vector), wild type EGFR (H1299-EGFR-WT) and EGFR with L858R mutant (H1299-EGFR-L858R).** *In situ* PLA images were shown in the Figure S2. Images from each slide with *in situ* PLA sample were acquired at 5 different fields with 2 z-axis images.
(DOC)

Table S2 **The antibody list for this study.**
(DOC)
