# Supplementary figures and images for: Protein Phosphorylation Profiling Using an In Situ Proximity Ligation Assay: Phosphorylation of AURKA-Elicited EGFR-Thr654 and EGFR-Ser1046 in Lung Cancer Cells
Source: PLoS One. 2013 Mar 8;8(3):e55657. doi: 10.1371/journal.pone.0055657 (PMC3592865; doi:10.1371/journal.pone.0055657)

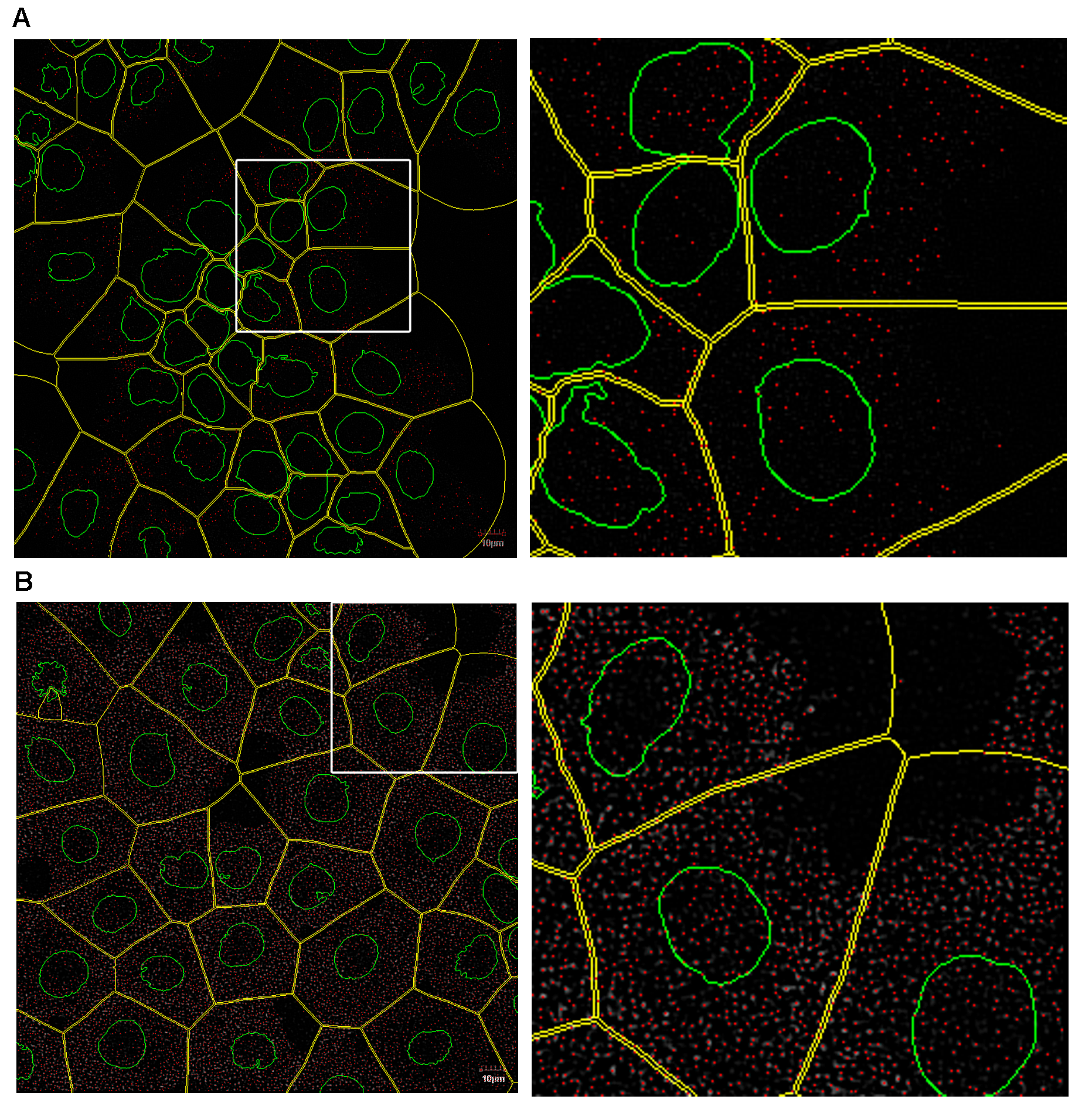

Supplement: Figure S1 — The calculation of in situ PLA signal in Figure 2A by BlobFinder. The raw images (RGB images) of Figure 2 were converted into gray-scale images (the white dots were in situ PLA signals). The in situ PLA signals, which were calculated by BlobFinder, were outlined with red dots. The white square of the left image was enlarged in the right. (A) The in situ PLA signal of pEGFR-Tyr1068 in A431 cells without EGF stimulation. (B) The in situ PLA signal of pEGFR-Tyr1068 in A431 with EGF stimulation. Green: nuclear. (TIF) [file pone.0055657.s001.tif]

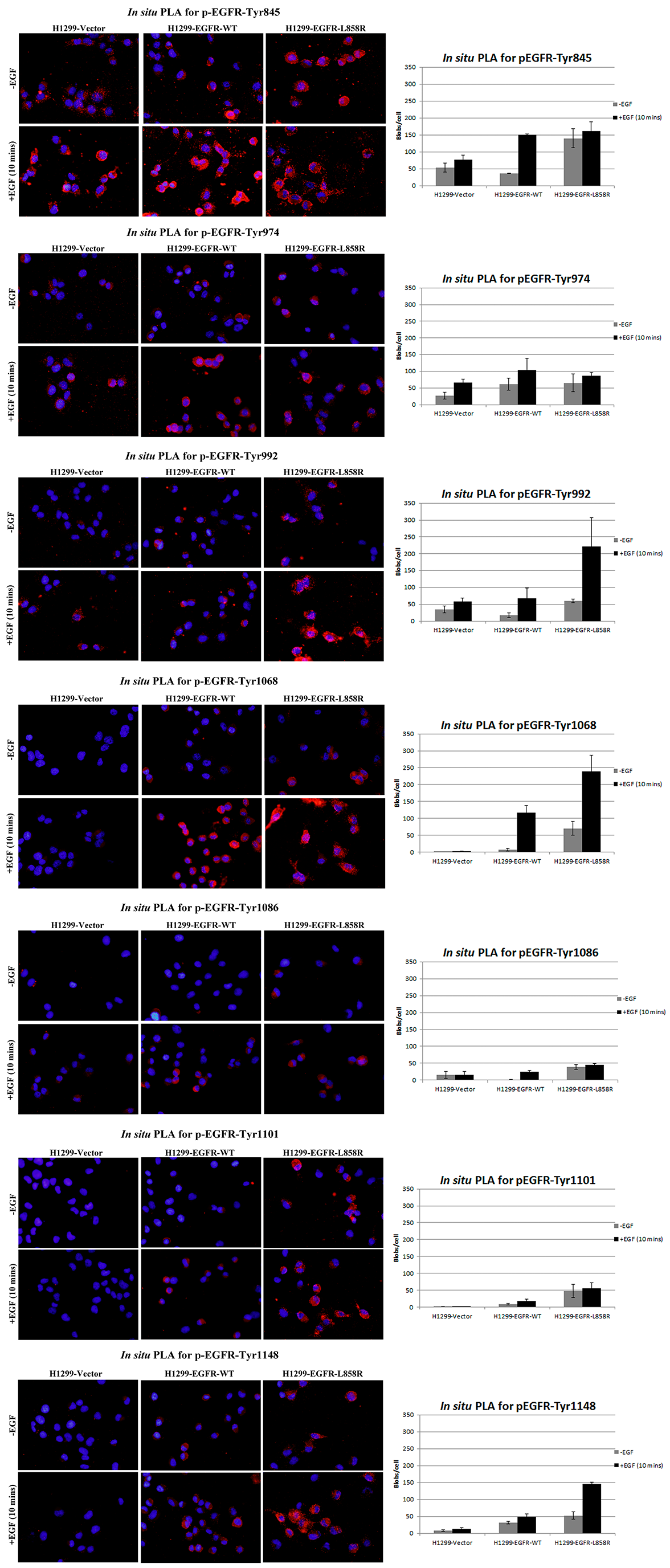

Supplement: Figure S2 — The determination of phosphorylation status of 9 phospho-EGFR sites by in situ PLA in the H1299 cells with expression of vector control (H1299-Vector), wild-type EGFR (H1299-EGFR-WT) and EGFR with L858R mutant (H1299-EGFR-L858R). The cells were stimulated with or without EGF (10 ng/ml) for 10 minutes followed by serum starvation for 16 hours. The quantification of in situ PLA signal was shown in the right. (TIF) [file pone.0055657.s002.tif]

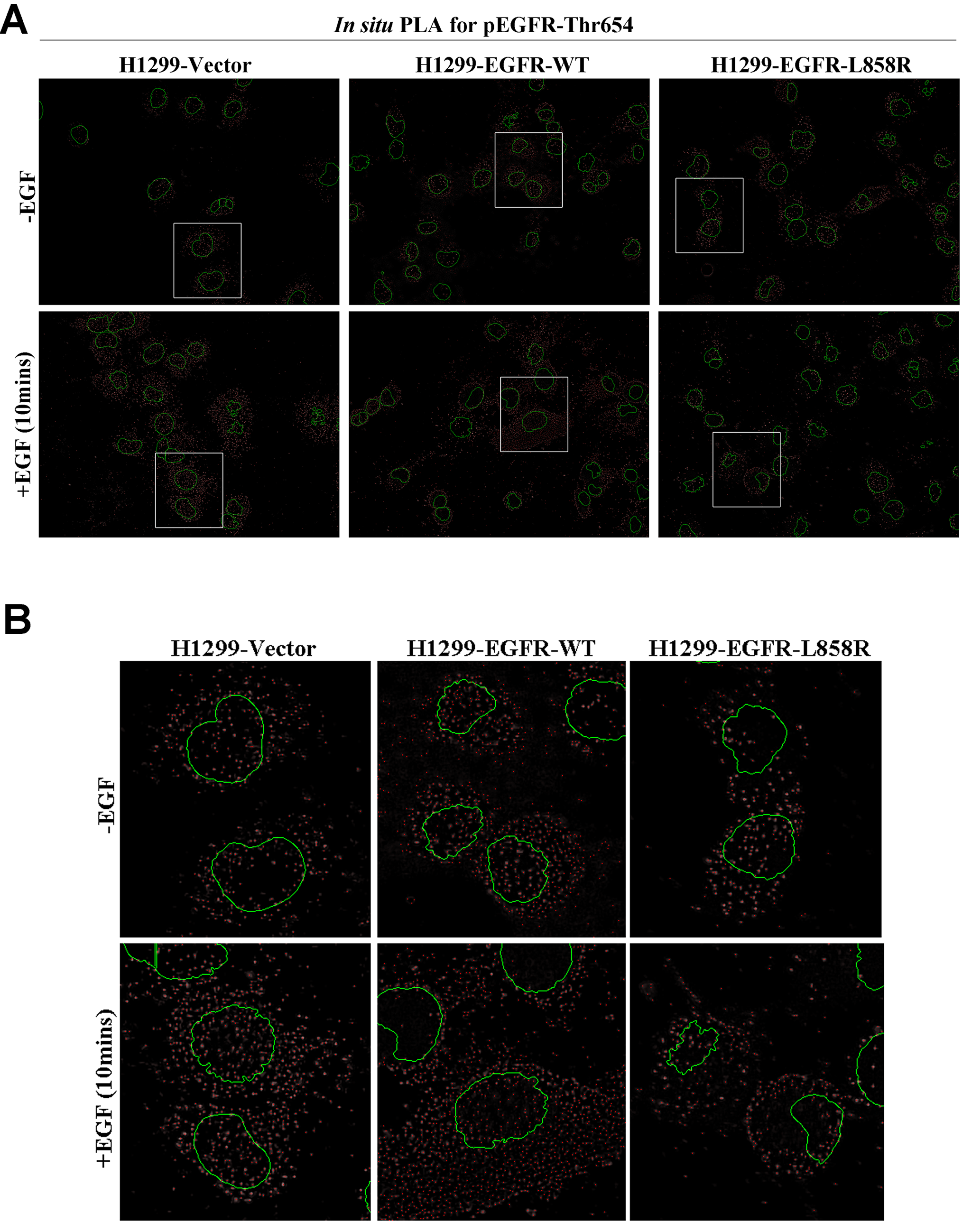

Supplement: Figure S3 — The calculation of in situ PLA signal of pEGFR-Thr654 in Figure 3B by BlobFinder. The raw images (RGB images) of Figure 3B were converted into gray-scale images (the white dots were in situ PLA signals). The in situ PLA signals, which were calculated by BlobFinder, were outlined with red dots. (A) The in situ PLA signals of pEGFR-Thr654. (B) The enlarged images of (A). Green: nuclear. (TIF) [file pone.0055657.s003.tif]

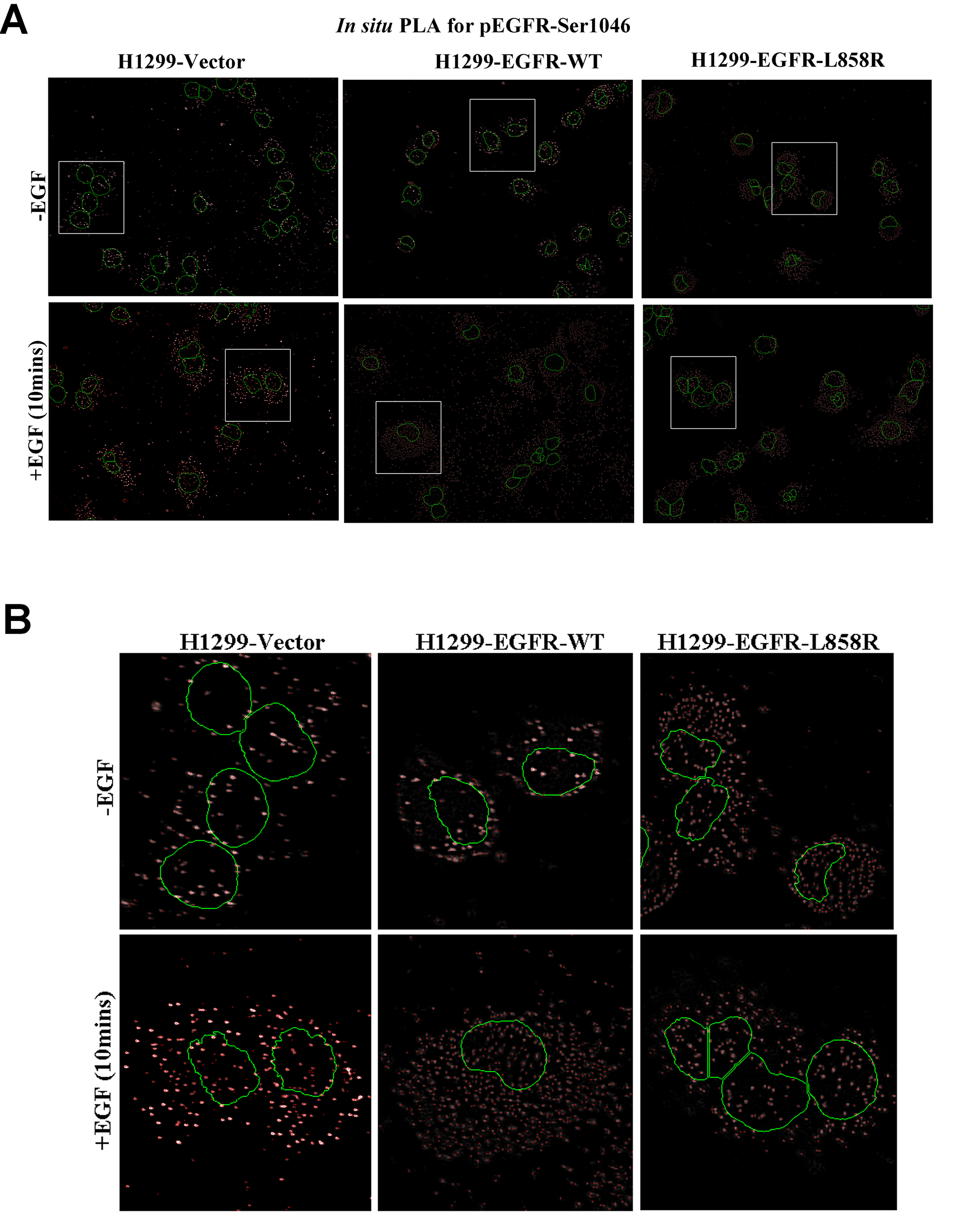

Supplement: Figure S4 — The calculation of in situ PLA signal pEGFR-Ser1046 in Figure 3C by BlobFinder. The raw images (RGB images) of Figure 3C were converted into gray-scale images (The white dots were in situ PLA signals). The in situ PLA signals, which were calculated by BlobFinder, were outlined with red dots. (A) The in situ PLA signals of pEGFR-Ser1046. (B) The enlarged images of (A). Green: nuclear. (TIF) [file pone.0055657.s004.tif]

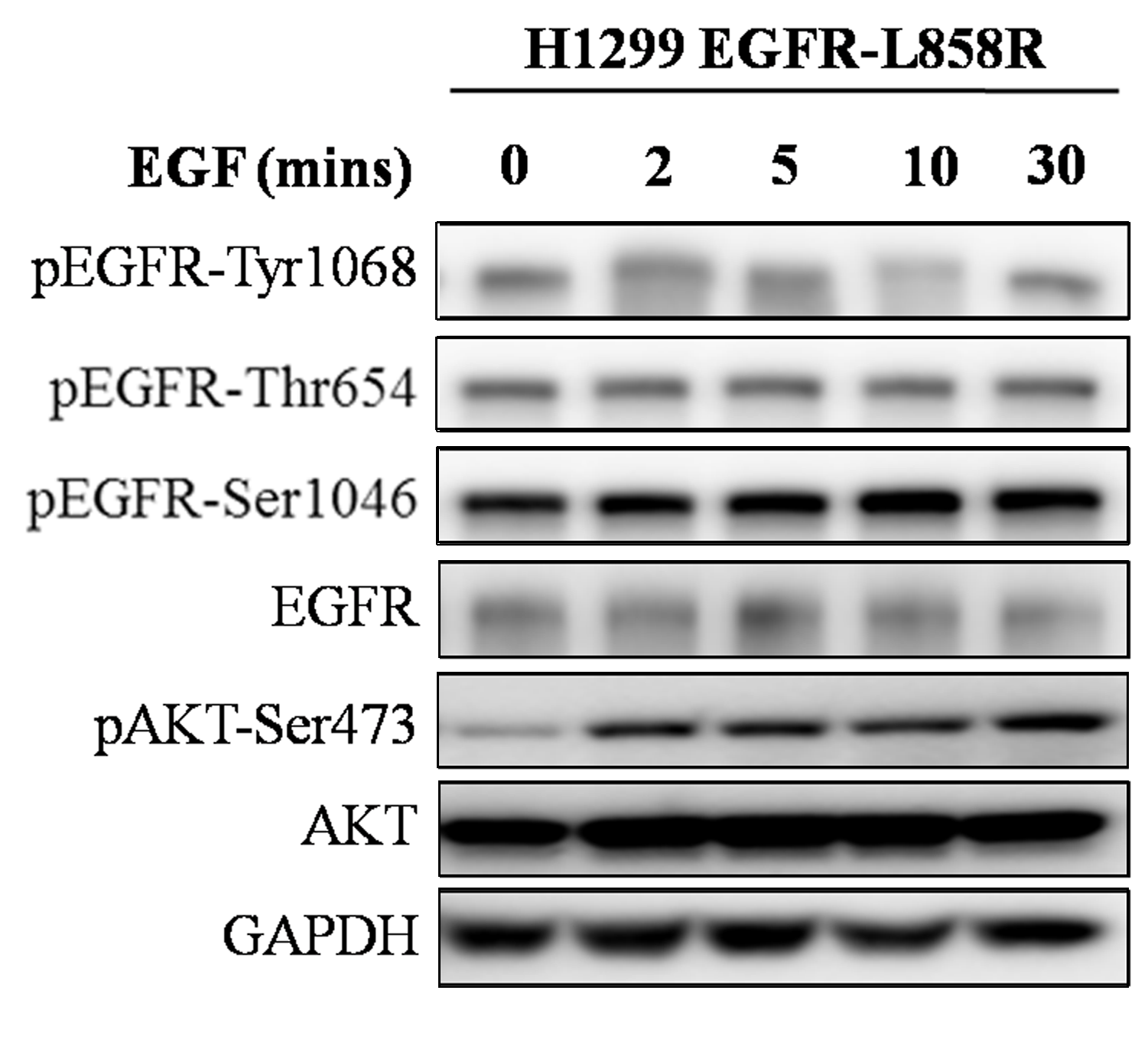

Supplement: Figure S5 — The phosphorylation kinetics of EGFR-Tyr1068, EGFR-Thr654, and EGFR-Ser1046 under the EGF (10 ng/ml) stimulation in H1299- EGFR -L858R cells. The phosphorylation kinetics of EGFR-Tyr1068, EGFR-Thr654, and EGFR-Ser1046 were not significantly changed when H1299-EGFR-L858R cells were treated with 10 ng/ml EGF for various time points. (TIF) [file pone.0055657.s005.tif]
