# Supplementary material for: Protein Phosphorylation Profiling Using an In Situ Proximity Ligation Assay: Phosphorylation of AURKA-Elicited EGFR-Thr654 and EGFR-Ser1046 in Lung Cancer Cells
Source: PLoS One. 2013 Mar 8;8(3):e55657. doi: 10.1371/journal.pone.0055657 (PMC3592865; doi:10.1371/journal.pone.0055657)
Supplement: Table S1 — The quantification of the phosphorylation status of 9 phospho-EGFR sites in the H1299 cells with expression of vector control (H1299-Vector), wild type EGFR (H1299-EGFR-WT) and EGFR with L858R mutant (H1299-EGFR-L858R). In situ PLA images were shown in the Figure S2. Images from each slide with in situ PLA sample were acquired at 5 different fields with 2 z-axis images. (DOC) [file pone.0055657.s006.doc]

| **EGFR phosphorylation site** | ***In situ* PLA signal of H1299-Vector (blobs/cell)^a^** | | ***In situ* PLA signal of H1299-EGFR-WT (blobs/cell)^a^** | | **Stimulation type in EGFR-wild type cells** | ***In situ* PLA signal of H1299-EGFR-L858R (blobs/cell)^a^** | | **Stimulation type in EGFR-L858R cells** |
| --- | --- | --- | --- | --- | --- | --- | --- | --- |
|  | -EGF | +EGF | -EGF | +EGF |  | -EGF | +EGF |  |
| pEGFR-Thr654 | 18.4 | 53.7 | 50.1 | 132.3 | EGF-dependent | 80.8 | 80.4 | EGF-independent |
| p-EGFR-Tyr845 | 43.56 | 77.11 | 36.59 | 151.22 | EGF-dependent | 140.28 | 161.67 | EGF-independent |
| p-EGFR-Tyr974 | 27.33 | 66.96 | 62.10 | 104.31 | EGF-dependent | 65.24 | 87.10 | EGF-independent |
| p-EGFR-Tyr992 | 35.10 | 58.74 | 18.38 | 68.61 | EGF-dependent | 60.44 | 221.21 | EGF-dependent |
| pEGFR-Ser1046 | 14.3 | 63.2 | 30.7 | 131.5 | EGF-dependent | 83.2 | 85.1 | EGF-independent |
| p-EGFR-Tyr1068 | 0.16 | 1.94 | 7.04 | 116.14 | EGF-dependent | 70.16 | 239.63 | EGF-dependent |
| p-EGFR-Tyr1086 | 15.40 | 15.45 | 1.49 | 24.27 | EGF-dependent | 38.89 | 45.54 | EGF-independent |
| p-EGFR-Tyr1101 | 0.97 | 0.25 | 8.84 | 18.42 | EGF-dependent | 47.89 | 54.91 | EGF-independent |
| p-EGFR-Tyr1148 | 8.51 | 13.17 | 31.79 | 48.31 | EGF-dependent | 52.81 | 146.45 | EGF-dependent |

**^a^**: The cells were stimulated with or without EGF (10 ng/ml) for 10 minutes followed by serum starvation for 16 hours.
