# Supplementary material for: Protein Phosphorylation Profiling Using an In Situ Proximity Ligation Assay: Phosphorylation of AURKA-Elicited EGFR-Thr654 and EGFR-Ser1046 in Lung Cancer Cells
Source: PLoS One. 2013 Mar 8;8(3):e55657. doi: 10.1371/journal.pone.0055657 (PMC3592865; doi:10.1371/journal.pone.0055657)
Supplement: Table S2 — The antibody list for this study. (DOC) [file pone.0055657.s007.doc]

| **Immunoblotting** | | | | | | | |
| --- | --- | --- | --- | --- | --- | --- | --- |
| **Rabbit polyclonal antibody** | | | **Company** | | **Catalog** | **Titration** | **Note** |
| anti-pEGFR-Thr654 | | | Abnova | | [PAB10949](http://www.abnova.com/products/products_detail.asp?Catalog_id=PAB10949) | 1:1000 |  |
| anti-pEGFR-Thr669 | | | Abnova | | [PAB10950](http://www.abnova.com/products/products_detail.asp?Catalog_id=PAB10950) | 1:1000 |  |
| anti-pEGFR-Ser671 | | | Abnova | | PAB0453 | 1:1000 |  |
| anti-pEGFR-Ser744 | | | Abnova | | PAB1662 | 1:1000 |  |
| anti-pEGFR-Tyr845 | | | Abnova | | PAB10953 | 1:1000 |  |
| anti-pEGFR-Tyr974 | | | Abnova | | [PAB0457](http://www.abnova.com/products/products_detail.asp?Catalog_id=PAB0457) | 1:1000 |  |
| anti-pEGFR-Tyr992 | | | Abnova | | [PAB1663](http://www.abnova.com/products/products_detail.asp?Catalog_id=PAB1663) | 1:1000 |  |
| anti-pEGFR-Tyr1045 | | | Cell Signaling | | 2237 | 1:1000 |  |
| anti-pEGFR-Ser1046 | | | Abnova | | [PAB10948](http://www.abnova.com/products/products_detail.asp?Catalog_id=PAB10948) | 1:1000 |  |
| anti-pEGFR-Tyr1068 | | | Cell Signaling | | 3777 | 1:1000 |  |
| anti-pEGFR-Tyr1086 | | | Abnova | | [PAB10952](http://www.abnova.com/products/products_detail.asp?Catalog_id=PAB10952) | 1:1000 |  |
| anti-pEGFR-Tyr1101 | | | Abnova | | [PAB0455](http://www.abnova.com/products/products_detail.asp?Catalog_id=PAB0455) | 1:1000 |  |
| anti-pEGFR-Tyr1148 | | | Abnova | | [PAB8085](http://www.abnova.com/products/products_detail.asp?Catalog_id=PAB8085) | 1:1000 |  |
| anti-pEGFR-Ser1166 | | | Abnova | | PAB0452 | 1:1000 |  |
| anti-pAURKA-Thr288 | | | Abcam | | ab58494 | 1:1000 |  |
| anti-GAPDH | | | Gene Tex | | 100118 | 1:6000 |  |
| **Rabbit monoclonal antibody** | | | **Company** | | **Catalog** | **Titration** | **Note^a^** |
| anti-pAKT-Ser473 | | | Cell Signaling | | 4060 | 1:1000 |  |
| anti-AKT | | | Cell Signaling | | 2938 | 1:1000 |  |
| **Mouse monoclonal antibody** | | | **Company** | | **Catalog** | **Titration** | **Note^a^** |
| anti-EGFR | | | Abcam | | ab62-100 | 1:1000 | The Epitope is 985-996 in the cytoplasmic domain of human EGFR |
| anti-AURKA | | | BD Bioscience | | 611083 | 1:1000 |  |
| anti-Flag-M2 | | | Sigma-Aldrich | | F1804 | 1:1000 |  |
| anti-Myc-Tag | | | Cell Signaling | | 2276 | 1:1000 |  |
| ***In situ* PLA** | | | | | | | |
| Pair | Rabbit polyclonal anti-pEGFR | Abnova | | See above | | 1:1200 |  |
|  | Mouse monoclonal anti-EGFR | Abcam | | ab62-100 | | 1:50 |  |
| Pair | Rabbit monoclonal anti-EGFR | Cell Signaling | | 4267 | | 1:100 |  |
|  | Mouse monoclonal anti-AURKA | Abnova | | H00006790-M01 | | 1:100 |  |
| **Immunohistochemistry** | | | | | | | |
| Rabbit polyclonal anti-pEGFR-Thr654 | | Abnova | | [PAB10949](http://www.abnova.com/products/products_detail.asp?Catalog_id=PAB10949) | | 1:300 |  |
| Rabbit polyclonal anti-pEGFR-Ser1046 | | Abnova | | [PAB10948](http://www.abnova.com/products/products_detail.asp?Catalog_id=PAB10948) | | 1:100 |  |
| Rabbit polyclonal anti-AURKA | | Sigma-Aldrich | | HPA002636 | | 1:400 |  |

**^a^**: The information of epitopes was obtained from the websites of antibody providers.
